# Identification and Characterization of a High Vancomycin-Resistant *Staphylococcus aureus* Harboring VanA Gene Cluster Isolated from Diabetic Foot Ulcer

**Published:** 2012

**Authors:** Anahita Dezfulian, Mohammad Mehdi Aslani, Mahvash Oskoui, Parisa Farrokh, Masumeh Azimirad, Hossein Dabiri, Mohammad Taghi Salehian, Mohammad Reza Zali

**Affiliations:** 1 *Research Centre of Gastroenterology and Liver Diseases (RCGLD) in Shahid Beheshti University, Tehran, Iran*; 2 *Department of Microbiology, Institute Pasteur of Iran, Tehran, Iran*

## Abstract

**Objective(s):**

*Staphylococcus aureus* is a common cause of human infection, and emergence of vancomycin-resistance* S. aureus* is a great concern for treatment of methicillin-resistant *S. aureus*,(MRSA) in recent years (MRSA). Here, we report the isolation of high-level VRSA.

**Materials and Methods:**

*S. aureus* was isolated from foot ulcer of a diabetic woman in Tehran, Iran. Antibiotic susceptibility was determined according to CLSI guidelines. VanA gene cluster PCR was carried out and PCR amplicon of *vanA* was sequenced.

**Results:**

*S. aureus* had high-level vancomycin-resistant (MIC 512 ≥ µg/ml). Patient's history revealed that VRSA isolate was acquired through community transmission. Only *vanA*, *vanR* and *vanS* genes were amplified in our isolate. Sequencing revealed that the *vanA* sequence had high similarity to the *vanA *sequence of *Tn1546*.

**Conclusion:**

Although VRSA infection continues to be rare, isolation of community–acquired VRSA is a significant issue and it needs the efforts of public health authorities.

## Introduction

*Staphylococcus aureus* is a common cause of hospital and community-acquired infections. Because of the spread of multidrug-resistant Gram-positive bacteria as well as methicilin-resistant *S. aureus* (MRSA), glycopeptide antibiotics, vancomycin and teicoplanin are used to treat severe staphylococcal infections (1). The first clinical vancomycin-resistance* S. aureus* (MIC ≥ 32 µg/mL) was reported from Michigan, USA in 2002 (2). It seems that the development of vancomycin-resistant enterococci (VRE) in 1988 led to the emergence of VRSA through acquisition of the VanA gene cluster from *Entercoccus *spp (3). The first detection of the VRSA in Iran was in 2007 (4) and this report describes clinical isolate of community–acquired vancomycin-resistant *S. aureus* from a diabetic patient in Iran with the vancomycin MIC 512 ≥ µg/ml.

## Materials and Methods

***Bacterial isolate***

A 51-year-old female with a history of diabetes mellitus was admitted to the Surgery Department of Taleghani Hospital, Tehran. Incision and drainage of the abscess was performed, and discharge was sent to the laboratories for microbiological and molecular investigation. Patient's medical records including antimicrobial drug history and recent bacterial infections were recorded. Isolate was identified based on colony morphology and standard biochemical tests. 

*S. aureus *ATCC 29213 and *Enterococcus faecalis* ATCC 29212 strains were used as vancomycin-susceptible controls. Vancomycin resistant *E. faecium* BM4147 was used as positive control. 

***Antimicrobial agents and MIC determination***

Antibiotic susceptibility was determined by disk diffusion on Mueller-Hinton agar (Merck) based on Clinical and Laboratory Standards Institute guidelines (5). The antibiotics (MAST Diagnostics Ltd. Merseyside, England) used for disc diffusion assays included vancomycin, teicoplanin, penicillin, oxacillin, ceftriaxone, erythromycin, clindamycin, amikacin, co-trimoxazole, chloramphenicol, amoxicillin, and imepenem. Minimum inhibitory concentration (MIC) of vancomycin (SERVA FEINBIOCHEMICA GmbH & Co., Germany) was determined by broth microdilution method according to CLSI guidelines (5). 

***Detection of vanA gene cluster by PCR***

VanA gene cluster (*vanR*, *vanS*, *vanH*, *vanA*, *vanX*, *vanY*, *vanZ*) PCR was carried out with previously published primers (6, 7). The DNA sequence of vanA was determined with an automated sequencer (ABI 377, Applied Biosystems [ABI]) using PCR product to determine sequences of the forward and reverse strands.

## Results

The isolate was identified as S. aureus and it was resistant to vancomycin, teicoplanin, penicillin, oxacillin, ceftriaxone, erythromycin, clindamycin, amikacin, co-trimoxazole, chloramphenicol, amoxicillin, and sensitive to imepenem. The isolate *showed high-level vancomycin* resistance (512 µg/ml).

Based on the resistance to glycopeptide antibiotics, the S. aureus* isolate expressed *VanA* phenotype*. 

Patient's history revealed that this isolate was a community–acquired VRSA. From VanA gene cluster, only PCR products of *vanA*, *vanR* and *vanS* were amplified from extracted DNA with expected size. 

DNA and the inferred amino acid sequences were compared using DNAsis (version 2.5; Hitachi**). **DNA sequence analysis revealed that this strain's *van*A sequence had high similarity to the *van*A sequence of *Tn1546* (M97297). The partial sequence of *vanA* gene has been submitted to GenBank (GQ273978). 

**Table 1:** Primers used in this study

primer	Sequence (5’ to 3’)	Size of PCR product (bp)*	Ref.
*van*R 1 *van*R 2	AGCGATAAAATACTTATTGTGGA CGGATTATCAATGGTGTCGTT	645	7
*van*S 1 *van*S 2	TTGGTTATAAAATTGAAAAATAA TTAGGACCTCCTTTTATC	1155	8
*van*H 1 *van*H 2	ATCGGCATTACTGTTTATGGAT TCCTTTCAAAATCCAAACAGTTT	943	7
*van*A 1 *van*A 2	ATGAATAGAATAAAAGTTGCAATAC CCCCTTTAACGCTAATACGAT	1029	7
*van*X 1 *van*X 2	ATGGAAATAGGATTTACTTT TTATTTAACGGGGAAATC	609	8
*van*Y 1 *van*Y 2	ATGAAGAAGTTGTTTTTTTTA TTACCTCCTTGAATTAGTAT	912	8
*van*Z 1 *van*Z 2	TTATCTAGAGGATTGCTAGC AATGGGTACGGTAAACGAGC	454	9

* Based on the sequence of Tn*1546* in *Enterococcus faecium* (GenBank accession no. M97297)

**Figure 1:**
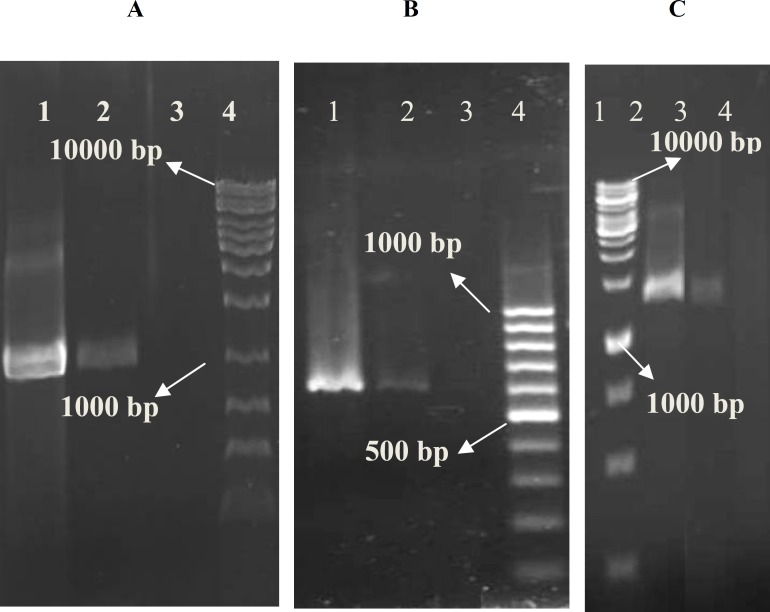
Specific PCR amplification products of the *vanA* gene cluster of *Staphylococcus aureus*. *van*A (A: lane 1,* van*A-positive control, BM4147; 2, isolated *S. aureus*; 3, *van*A-negative control, ATCC 29213; 4, molecular weight marker, 1kb); *van*R (B: lane 1,* van*A-positive control, BM4147; 2, isolated *S. aureus*; 3,* van*A-negative control, ATCC 29213; 4, molecular weight marker, 100 bp) and *van*S (C: lane 1, molecular weight marker, 1kb; 2,* van*A-positive control, BM4147; 3, isolated *S. aureus*; 4,* van*A-negative control, ATCC 29213)

## Discussion

There are limited reports about isolation of VRSA from clinical specimens in all over the world and among these isolates a few of them were community–acquired VRSA (8**)**. To the best of our knowledge, it is the first report of community–acquired of vancomycin and methicillin-resistant *S. aureus* in Tehran, Iran. 

According to the *in vitro* transfer of *vanA* gene from enterococci to *S. aureus *(8**)**, we suspect the possibility of transformation of vancomycin resistance gene (*vanA*) from VRE to *Staphylococci *spp.

In this study there were significant differences between previously described VanA gene cluster among enterococci and our isolate. Although the* vanA*, *vanR*, *vanS*, *vanH* and *vanX* genes are essential for the expression of VanA phenotype, according to the previous study (9), the difficulties with amplification of* vanH* and *vanX* in the *vanA* gene cluster may be due to disruptions of these regions with insertion sequences (ISs). 

Prevention of emergence and transmission of VRSA in each community is needed. So, use of proper infection-control practices, appropriate antimicrobial agent management, maintaining a clean environment and increased awareness can control the spread of antimicrobial-resistant microorganisms, including VRSA.

## Conclusions

This report describes a community–acquired and multidrug-resistant S. aureus* isolated from a diabetic patient in* Tehran, Iran.* Among antibiotics used in this study, co-resistance to *oxacillin and vancomycin is a critical issue because vancomycin is the first-line antimicrobial agent for the treatment of infection with MRSA.
